# Case of Monstrosity

**Published:** 1845-11

**Authors:** 


*Case of Monstrosity.* By M. Nicolas.—M. Nicolas presented to the Academy of Medicine the body of a new-born infant, in which the bones of the upper portion of the cranium were wanting. A considerable portion of the brain escaped during its delivery, yet the child lived an hour, and uttered strong cries.—*Ibid, from Bul. de l'Acad. Roy. de Med.*

